# Editorial: Insect Physiology Aspects of Environmentally Friendly Strategies for Crop Pests and Insect Vectors Control

**DOI:** 10.3389/fphys.2022.905301

**Published:** 2022-05-11

**Authors:** Ana Claudia Amaral Melo, Guenter Arthur Schaub, Marcelo Salabert Gonzalez, Norman Arthur Ratcliffe

**Affiliations:** ^1^ Chemistry Institute, Federal University of Rio de Janeiro, Rio de Janeiro, Brazil; ^2^ National Institute of Science and Technology - Molecular Entomology, Rio de Janeiro, Brazil; ^3^ Chair of Evolutionary Ecology and Animal Biodiversity, Working Group for Zoology and Parasitology, Ruhr-Universität Bochum, Bochum, Germany; ^4^ Department of General Biology, Institute of Biology, Fluminense Federal University, Niterói, Brazil; ^5^ Postgraduate Program in Science and Biotechnology, Fluminense Federal University, Niterói, Brazil; ^6^ Postgraduate Program in Applied Physics, Institute of Physics, Federal University of Rio de Janeiro, Rio de Janeiro, Brazil; ^7^ Department of Biosciences, Swansea University, Swansea, United Kingdom

**Keywords:** insect physiology, control of arthropod populations, crop pests, insect vectors, environmentally friendly strategies

Enhancing our knowledge concerning the control of invertebrate populations, mainly of agricultural pests and disease vectors, is nowadays one of the World’s greatest scientific challenges. In the context of environmentally friendly interventions to control crop pests and insect vectors, this Research Topic focuses particularly relevant scientific articles on aspects of arthropod physiology. These include insect responses to plant and animal volatiles which enhance attraction and repellence in Tsetse flies (Mireji et al.), potential genes and neuropeptides for reproductive regulation in *Bombyx mori* (Xu et al.) and *Rhipicephalus sanguineus* (Xiong et al.), a meta-analysis method to evaluate insecticide resistance (Wang et al.), screening of plant metabolites acting as physiologically active compounds in *Rhipicephalus microplus* (Mattos et al.), and a review concerning delivery of dsRNA molecules within the cells of targeted pest control insects (Swevers et al.). Together, these research papers present recent advances in understanding the manipulation of both invertebrate physiology and ecological elements using refined molecular approaches and represent innovative perspectives in the field of environmentally friendly strategies for the control of important arthropod populations, avoiding the use of insecticides toxic to animals and humans.

